# Nævus unius lateris: un nævus déroutant (à propos d´un cas)

**DOI:** 10.11604/pamj.2021.39.286.28932

**Published:** 2021-08-31

**Authors:** Sara Kerroum, Ibtissam Boubnane, Mariame Meziane, Karima Senouci

**Affiliations:** 1 Centre Hospitalier Universitaire Ibn Sina, Faculté de Médecine et de Pharmacie, Université Mohamed V, Rabat, Maroc

**Keywords:** Nævus unius lateris, nævus verruqueux épidermique, dysembryoplasie, à propos d’un cas, Naevus unius lateris, verrucous-epidermal-nevus, dysembryoplasia, case report

## Abstract

Le nævus unius lateris est un hamartome congénital rare dérivé de l´ectoderme. Il est considéré comme une variante verruqueuse du nævus épidermique. En raison de sa distribution unilatérale étendue, il est fréquemment associé à des anomalies neurologiques, musculo-squelettiques, auditives et visuelles. Nous rapportons dans cet article, le cas d´un enfant de 9 ans présentant un nævus unius lateris associé à des anomalies neurologiques et oculaires.

## Abstract (en)

Naevus unius lateris is a rare congenital hamartoma, originating from the ectoderm. It is considered as a verruquous variant of the epidermal naevus. Given its extensive unilateral distribution, it is frequently associated with neurological, musculoskeletal, auditory and visual abnormalities. We here report the case of a 9-year-old child with nevus unius lateris associated with neurological and eye abnormalities.

## Introduction

L´une des plus intéressantes et certainement les plus insolites variétés de nævus est le nævus unius lateris. Cette dysembryoplasie est caractérisée par un développement extensif unilatéral de l´épiderme et peut être associée à des manifestations neurologiques, musculo-squelettiques, auditives et visuelles [1]. Peu de cas ont été signalés jusqu´à présent dans la littérature.

## Patient et observation

**Information sur le patient:** un enfant de 9 ans, d´origine marocaine, sans notion de consanguinité suivi en neurologie pour épilepsie et troubles du comportement sous Acide Valproïque 500mg/j et en ophtalmologie pour opacité cornéenne et sans antécédents familiaux similaires.

**Figure 1:**
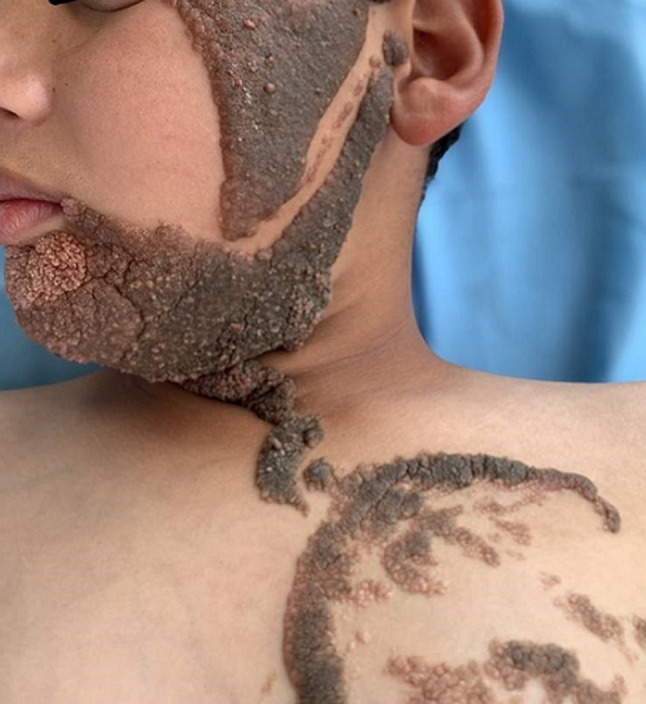
atteinte du visage

**Figure 2:**
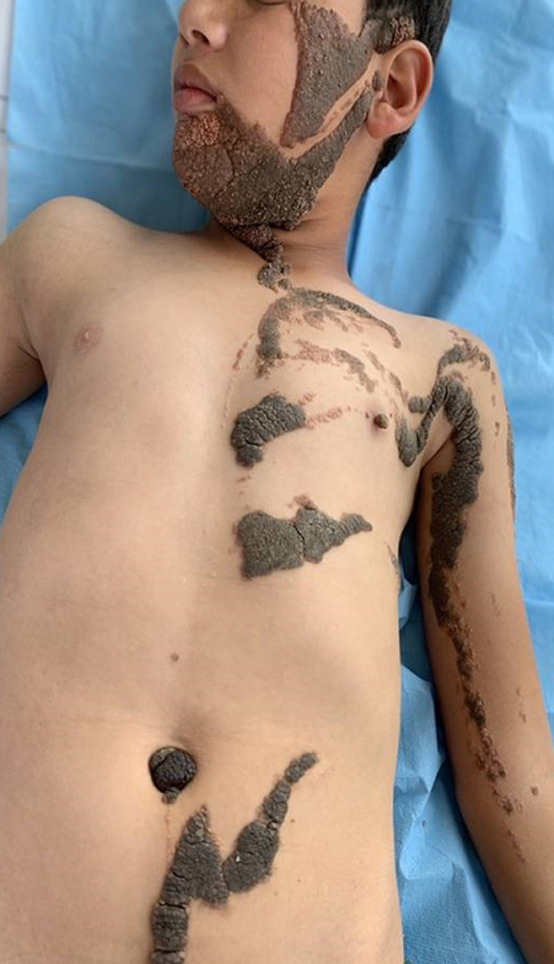
atteinte du corps

**Figure 3:**
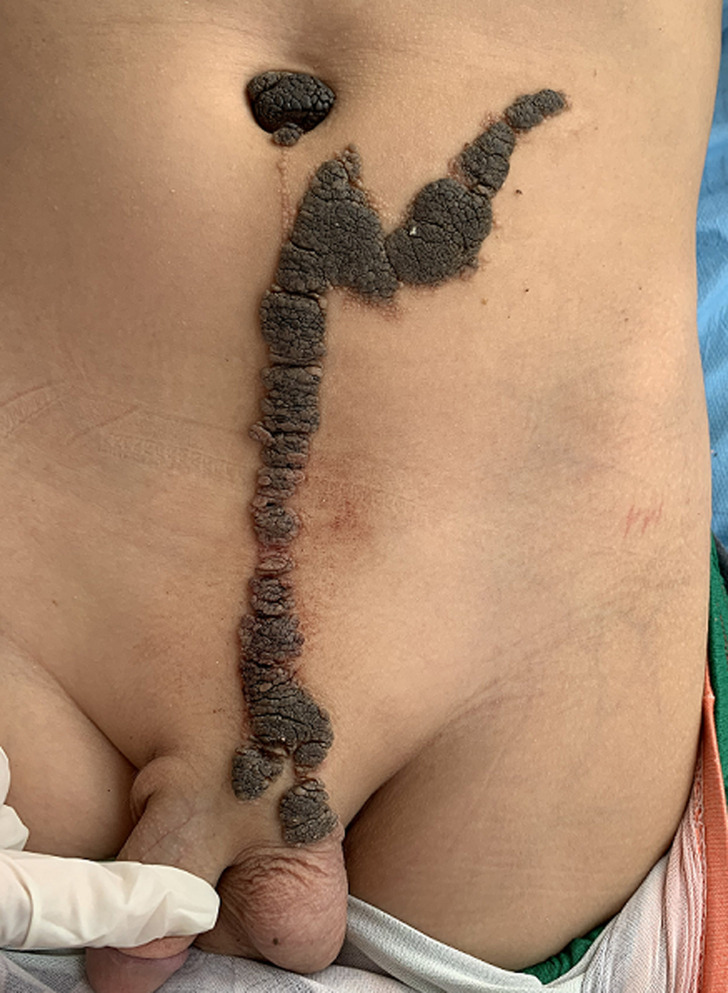
atteinte de la muqueuse génitale

**Figure 4:**
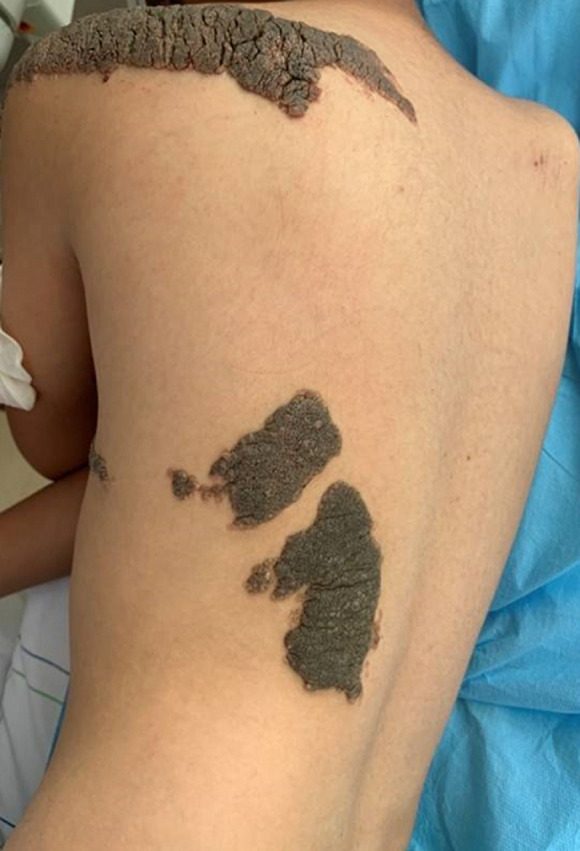
lésions ne dépassant pas la ligne médiane

Constatation clinique: cet enfant présentait des formations verruqueuses, hyperpigmentées intéressant l´hémicorps gauche au niveau: des 2/3 inférieurs du visage (Figure 1), le cou, l´épaule, le tronc, l´avant-bras, l´abdomen (Figure 2) et atteignant la muqueuse génitale (Figure 3). Les lésions décrites étaient présentes à la naissance et ont progressivement augmenté de taille et d´épaisseur sans dépasser la ligne médiane (Figure 2, Figure 4). La courbe de croissance était normale par rapport à l´âge et l´examen ostéo-articulaire ainsi que le reste de l´examen clinique étaient sans particularités.

**Intervention thérapeutique:** l´enfant est candidat à un traitement par laser CO2.

**Suivi et résultats:** le patient a bénéficié de 3 séances de laser CO2 avec une légère réduction de la taille des lésions.

## Discussion

Le nævus verruqueux épidermique a une prévalence estimée dans la population générale à 1/1000, cependant sa variante nævus unius lateris ne représente que 0,01% de ce total [2]. Le naevus unius lateris apparaît à la naissance ou dans les premières années de vie et persiste tout au long de la vie. L´aspect clinique le plus courant est la présence de formations verruqueuses, hyperpigmentées et hyperkératosiques [3] comme c´est le cas de notre patient.

La localisation la plus fréquente est sur le tronc et les membres avec un arrêt soudain des lésions au niveau de la ligne médiane. Cette entité clinico-pathologique est généralement associée à des manifestations neurologiques, des troubles musculo-squelettiques, auditifs et visuels, c´est le cas de notre patient qui présente une épilepsie, troubles du comportement et une opacité cornéenne. La clinique est la pierre angulaire du diagnostic, néanmoins dans certains cas une biopsie peut-être nécessaire pour confirmer le diagnostic. Concernant l´évolution, la majorité des lésions restent asymptomatiques, mais le risque de surinfection bactérienne existe surtout si les lésions se situent dans des zones de frottement et de friction. Le traitement des lésions étendues est difficile. Les techniques chirurgicales [4], la cryothérapie, le laser CO2, la thérapie photodynamique, le calcipotriol et les rétinoïdes locaux et systémiques peuvent tous être utilisés. Les résultats sont variables et les récidives sont fréquentes. Notre patient est candidat à un traitement par laser CO2.

L´étiopathogénie de cette entité clinique reste inconnue, cependant, si le nævus suit les lignes de Blaschko, il est considéré comme un mosaïsme. Les cas familiaux sont rares. Notre patient n´avait aucun antécédent familial similaire.

**Point de vue du patient et de sa famille:** durant les séances de traitement le patient et sa famille étaient satisfaits des soins reçus et optimistes quant à l´évolution thérapeutique.

**Consentement éclairé:** le patient et son père ont été informés du rapport de cas, des raisons pour lesquelles son cas était particulier et de l'intérêt des auteurs à publier son cas. Ils ont volontairement donné leur consentement éclairé pour permettre aux auteurs d'utiliser ses photos pour ce rapport de cas.

**Consentement du patient:** le consentement éclairé a été obtenu du patient et de sa famille pour que nous utilisions les images.

## Conclusion

Nous rapportons un cas de nævus unius lateris. Cette dysembryoplasie congénitale est rarement décrite et est dans la majorité des cas associée à d´autres pathologies, d´où l´importance cruciale d´un diagnostic précoce et un suivi régulier des patients atteints.

## References

[1] Kharel Narine, Litzel Carrera (2019). Nevus Unius Lateris: a case report. Cureus.

[2] Pack George T, Sunderland Douglas A (1941). Naevus Unius Lateris. Arch Surg.

[3] Happle Rudolf, Metze Dieter, Casano Angel Vera (2010). Naevus Lentiginosus linearis: a distinct skin disorder. Acta Derm venereol.

[4] Jesus AA, Jorge OC, Aide LOP, Daniel VVC, Osvaldo VM (2018). Nevus Unius lateris: electrofulguration as a therapeutic approach. J Dermatol.

